# Mucinous cystic neoplasm of the pancreas activated during pregnancy

**DOI:** 10.1186/s40792-014-0012-2

**Published:** 2015-02-10

**Authors:** Keisuke Kosumi, Hiroshi Takamori, Daisuke Hashimoto, Hiroshi Tanaka, Shinya Abe, Osamu Nakahara, Kei Horino, Hideo Baba

**Affiliations:** Department of Gastroenterological Surgery, Graduate School of Medical Sciences, Kumamoto University, 1-1-1 Honjo, Chuo-ku, Kumamoto 860-8556 Japan

**Keywords:** Mucinous cystic neoplasm, Pancreas, Activation, Pregnancy

## Abstract

The characteristic histological feature of pancreatic mucinous cystic neoplasm (MCN) is ovarian-like stroma (OS) underlying the epithelium and existence of estrogen receptors and progesterone receptors in the nucleus of OS. We experienced a case of pancreatic MCN which was activated during pregnancy and confirmed the existence of estrogen receptors and progesterone receptors. In cases with potential factors for malignancy, surgical resection of MCN may be needed during pregnancy. On the other hand, in cases without these, as female sex hormones may have an influence on the behavior of pancreatic MCN during pregnancy, the timing of surgery should be decided on a case-by-case basis, taking into consideration the status of the malignancy, the stage of the pregnancy, and the condition of the mother and fetus.

## Background

A mucinous cystic neoplasm (MCN) is relatively rare, accounting for about 8% of resected cystic lesions of the pancreas [1]. And the characteristic histological feature is ovarian-like stroma (OS) underlying the epithelium [2]. As with an ovarian MCN, estrogen receptors (ER) and progesterone receptors (PgR) are expressed in OS of pancreatic MCN [3], indicating that female sex hormones may have an influence on the behavior of pancreatic MCN, especially during pregnancy. We experienced a patient with pancreatic MCN which was activated during the pregnancy. Herein, we introduce the case, together with a literature review of MCN during pregnancy.

## Case presentation

**Figure 1:**
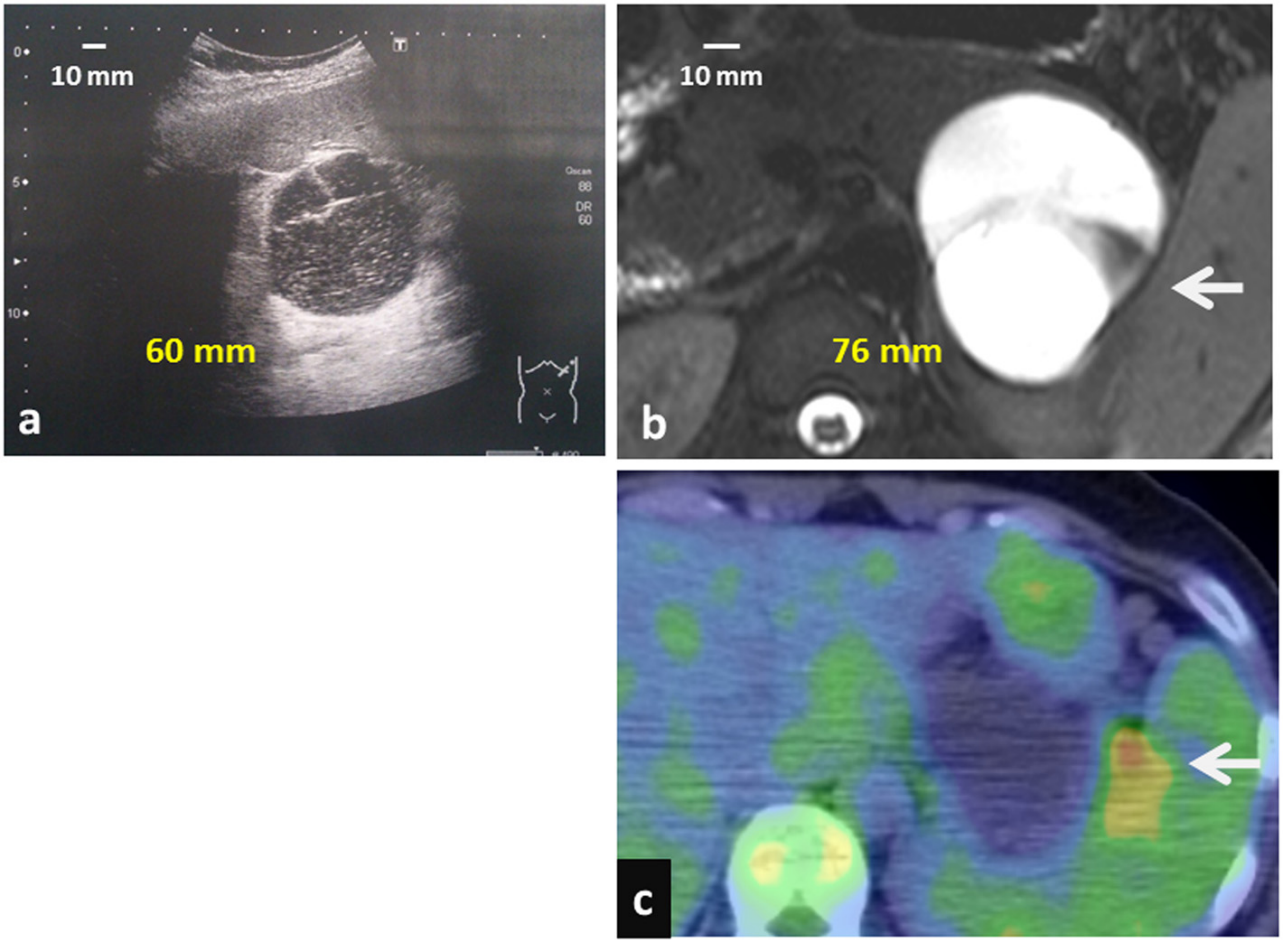
**Imaging studies. (a)** A 60-mm cystic mass was detected in the body and tail of the pancreas without a mural nodule or thickening of the wall by abdominal ultrasound (during the fourth month of gestation). **(b)** Magnetic resonance imaging (MRI) showed a 76 × 69-mm cystic tumor (arrow), in which the contents were hyperintense in T2-weighted imaging. There were septa in the tumor, and one part of it was thickened and enhanced. **(c)** Fluorodeoxyglucose positron emission tomography-computed tomography (FDG-PET/CT) showed high uptake (arrow) in part of the cystic tumor (SUVmax 3.03).

A 33-year-old woman who was 4 months pregnant complained of left back pain. Abdominal ultrasound revealed a 60-mm cystic mass in the body and tail of the pancreas without a mural nodule or thickening of the wall (Figure 1a). The serum cancer antigen (CA) 19-9 level was elevated, at 92 U/mL (normal level is <37 U/mL), and then rapidly rose to 2,157 U/mL just before delivery.

**Figure 2:**
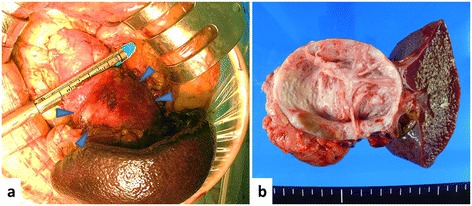
**Intraoperative findings and macroscopic observations. (a)** A smooth cystic tumor occupied the body and tail of pancreas (arrowheads indicating the tumor). Distal pancreatectomy was performed. **(b)** The cut surface of the tumor showing a large cyst without solid components.

**Figure 3:**
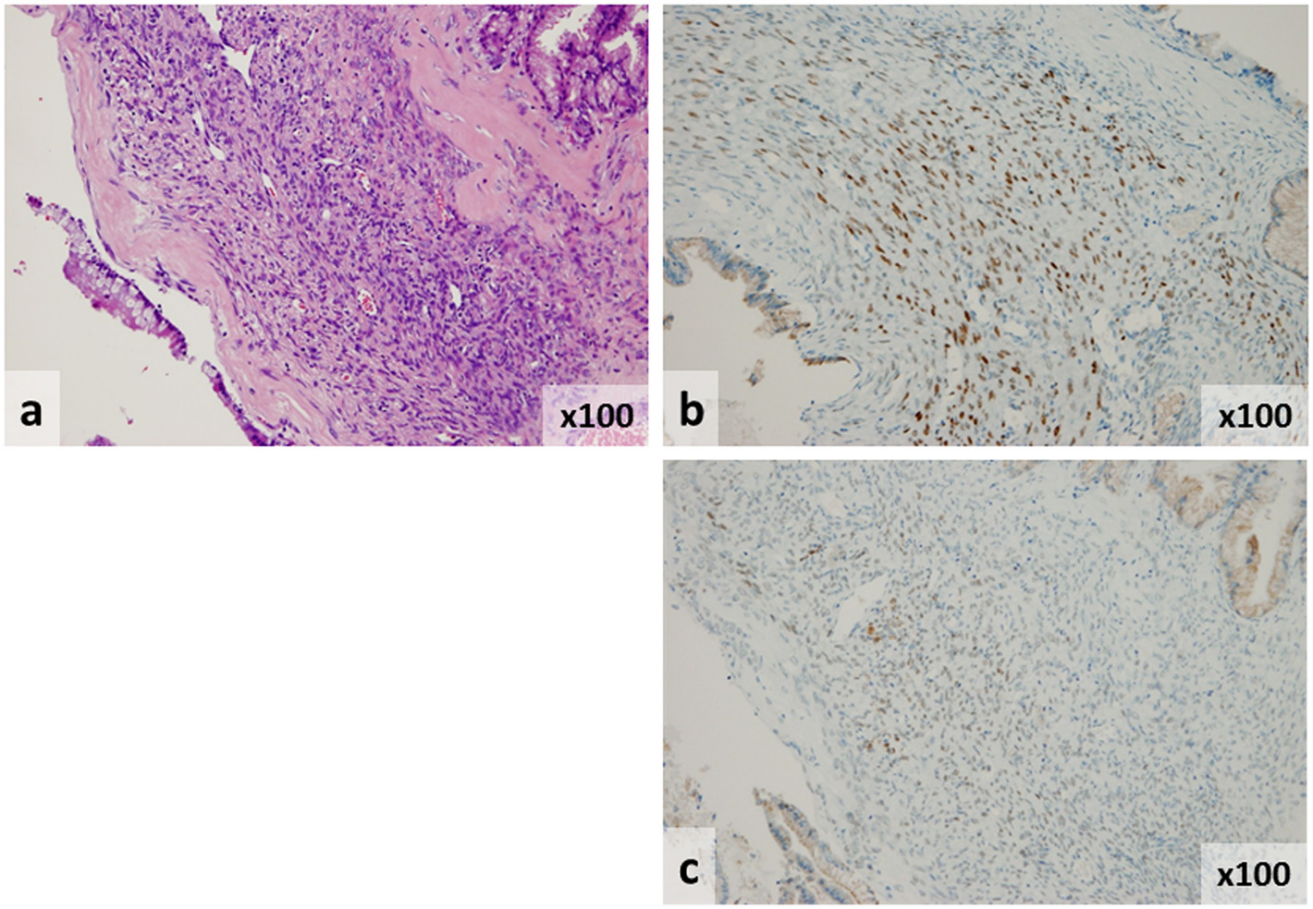
**Analysis of pancreatic mucinous cystic adenoma by immunohistochemistry. (a)** The cyst wall was lined with benign mucinous columnar epithelium underlying OS (Hematoxylin and eosin, ×100). **(b)** Immunohistochemical studies showed partial positive staining for estrogen receptors in the nucleus of ovarian-type stroma (×100). **(c)** Immunohistochemical studies showed partial positive staining for progesterone receptors in the nucleus of ovarian-type stroma (×100).

The patient's antenatal course was uneventful, and the delivery was normal. After delivery, magnetic resonance imaging revealed that the cystic tumor was 76 mm in diameter, with a thickening septum seen on T2-weighted imaging (Figure 1b). In addition, fluorodeoxyglucose positron emission tomography-computed tomography (FDG-PET/CT) showed an abnormally high uptake in part of the cystic tumor (Figure 1c). As FDG-PET/CT imaging indicated that malignancy could not be excluded, the patient underwent distal pancreatectomy on the 14th day after delivery (Figure 2a,b). Microscopically, the cyst wall was lined with benign mucinous columnar epithelium underlying OS (Figure 3a). In addition, immunohistochemical analysis showed that both ER and PgR were partially positive in the nucleus of OS (Figure 3b,c).

All pancreatic MCNs should be resected because they are considered as having malignant potential [4]. As increase in tumor size, mural nodules, and eggshell calcification predict malignant MCN [5], the ideal strategy is to remove the MCN surgically before these predictors develop. However, it is difficult to determine the best timing for surgery when pancreatic MCN is detected during pregnancy. There are several points of view. First, an accurate diagnosis of malignancy is difficult, especially for borderline cases. Second, it is necessary to consider the effects of surgery on the mother and the fetus.

We analyzed the clinical characteristics of MCNs detected during pregnancy according to data extracted from our case and previous reports [6-24], as well as our own experience (Table 1). In the reports reviewed, 12 patients underwent surgery during pregnancy, and neither miscarriage nor operation-related death occurred in any of the patients. Eight patients underwent operation after delivery. Regarding histopathological diagnosis, 14 patients were diagnosed with benign MCN, and 6 patients, 3 patients underwent operation during pregnancy and 3 patients after delivery, were diagnosed with adenocarcinoma. Among the six patients with adenocarcinoma, at least four patients were alive 1 year after resection.

**Table 1:** **Literatures review of clinical characteristics of mucinous cystic neoplasm during pregnancy**

**Author [reference number]**	**Age**	**Maximum diameter of tumor (cm)**	**Diameter of tumor growth during pregnancy (mm)**	**CA19-9 (U/mL)**	**Timing of operation**	**Histological diagnosis**	**ER/PgR**	**Prognosis (years)**
Smithers [6]	33	10	NA	NA	During pregnancy	Adenocarcinoma	NA	NA
Baiocchi [7]	29	NA	NA	NA	After delivery	Adenocarcinoma	NA	Alive (2)
Olsen [8]	25	5	0	NA	During pregnancy	Benign	NA	
Ganepola [9]	37	12	65	NA	During pregnancy	Benign	+/+	
Kato [10]	33	22	+	NA	During pregnancy	Benign	+/+	
Matsunaga [11]	28	20	+	NA	During pregnancy	Adenocarcinoma	−/+	NA
Fernandez [12]	26	15	NA	NA	During pregnancy	Benign	NA	
Herring [13]	34	20	80	NA	During pregnancy	Adenocarcinoma	+/+	Alive (NA)
Ozden [14]	32	15	NA	NA	After delivery (emergency cesarean)	Adenocarcinoma	−/−	Alive (1)
Ishikawa [15]	33	18	60	NA	After delivery	Benign	−/−	
Ikuta [16]	30	18	NA	Normal	After delivery (abortion)	Benign	+/+	
Hakamada [17]	38	14	40	NA	During pregnancy	Benign	NA/+	
Wiseman [18]	32	15	NA	NA	During pregnancy	Benign	+/+	
Brown [19]	38	10	NA	NA	During pregnancy	Benign	NA	
Shirakawa [20]	34	19	0	Elevated	After delivery	Benign	+/+	
Asciutti [21]	31	8	65	214	After delivery	Benign	NA	
Nagamura [22]	32	11	NA	4,750	After delivery (emergency cesarean)	Adenocarcinoma	−/+	Alive (3)
Boyd CA [23]	21	17.2	0	NA	During pregnancy	Benign	NA	
Tsuda [24]	28	14	10	10	During pregnancy	Benign	+/+	
Present case	33	7.6	16	2,157	After delivery	Benign	+/+	

CA19-9, cancer antigen 19–9; ER, estrogen receptor; NA, not available; PgR, progesterone receptor.

Importantly, in our case, we also confirmed the existence of ER and PgR. In addition, we confirmed rapid growth of MCN during pregnancy in nine patients, and at least seven patients of them showed positive staining for ER or PgR. Interestingly, Tanaka et al. reported a case of MCN developing during continuous hormone replacement therapy after hysterectomy. These findings suggested that the existence of ER and PgR might contribute to the activation of MCN during pregnancy.

Although the cutoff value of size and the mural nodule diameter for predicting malignancy has not been determined, it was generally reported that factors for predicting malignant MCN were large size and the existence of mural nodes [25]. In our case, we detected not only the two signs of malignant MCN but also an elevated CA19-9 level from 92 to 2,157 U/mL. A CA19-9 concentration >37 U/mL had a positive predictive value of 95.7% for potentially malignant lesions, but only showed a sensitivity of 35.8% [26]. In Table 1, five of the six patients showed the elevation of serum CA19-9 level, and the serum CA19-9 level of the patient with adenocarcinoma was remarkably higher. However, we could not find any relationships between the expression of hormone receptors and CA19-9 level, so further studies are necessary to validate the relationship between tumor markers and clinicopathological features including malignancy and hormone receptors.

In cases with potential factors for malignancy like large size and thickening septum, surgical resection of MCN may be needed. On the other hand, no findings of malignancy may enable the extension of surgical resection until delivery. In evaluating the possibility of malignant status of a MCN, consideration must be given to the stage of pregnancy and the condition of the mother and the fetus, and the timing of surgery should be decided on a case-by-case basis. Moreover, in view of the existence of ER and PgR, careful observation is necessary concerning the growth and progression of MCN.

## Conclusions

In cases with potential factors for malignancy, surgical resection of MCN may be needed during pregnancy. On the other hand, in cases without these, as female sex hormones may have an influence on the behavior of pancreatic MCN during pregnancy, the timing of surgery should be decided on a case-by-case basis, taking into consideration the status of the malignancy, the stage of the pregnancy, and the condition of the mother and fetus.

## Consent

Written informed consent was obtained from the patient for publication of this case report and any accompanying images. A copy of the written consent is available for review by the Editor-in-Chief of this journal.

## Abbreviations

CA19-9: cancer antigen 19-9
ER: estrogen receptors
FDG-PET/CT: fluorodeoxyglucose positron emission tomography-computed tomography
MCN: mucinous cystic neoplasm
MRI: magnetic resonance imaging
NA: not available
OS: ovarian-like stroma
PgR: progesterone receptors
SUVmax: maximum standardized uptake value
